# Real-world alcohol cue exposure and drinking risk in alcohol use disorder

**DOI:** 10.1038/s41398-026-04506-4

**Published:** 2026-10-08

**Authors:** Ali Ghadami, Abhijit Sreepada, Iris Reinhard, Marvin Ganz, Justin Boehmer, Kanya Meyer-Degering, Henrik Walter, Jamila Andoh, Bernd Lenz, Patrick Bach, Falk Kiefer, Emanuel Schwarz, Markus Reichert, Ulrich W. Ebner-Priemer, Andreas Meyer-Lindenberg, Christine Heim, Heike Tost

**Affiliations:** 1 Department of Psychiatry and Psychotherapy, Central Institute of Mental Health, University of Heidelberg, Medical Faculty Mannheim, Mannheim, Baden-Württemberg Germany; 2 German Center for Mental Health (DZPG), Partner Site Mannheim-Heidelberg-Ulm, Germany; 3 Core Facility Biostatistics, Central Institute of Mental Health, University of Heidelberg, Medical Faculty Mannheim, Mannheim, Baden-Württemberg Germany; 4 Charité-Universitätsmedizin Berlin, Corporate Member of Freie Universität Berlin and Humboldt-Universität zu Berlin, Institute of Medical Psychology, Berlin, Germany; 5 Charité-Universitätsmedizin Berlin, Corporate Member of Freie Universität Berlin and Humboldt-Universität zu Berlin, Department of Psychiatry and Psychotherapy (CCM), Berlin, Germany; 6 German Center for Mental Health (DZPG), Partner Site Berlin-Potsdam, Germany; 7 Department of Addictive Behavior and Addiction Medicine, Central Institute of Mental Health, Medical Faculty Mannheim, Heidelberg University, Mannheim, Baden-Württemberg Germany; 8 Hector Institute for Artificial Intelligence in Psychiatry, Central Institute of Mental Health, Medical Faculty Mannheim, Heidelberg University, Mannheim, Baden-Württemberg Germany; 9 Department of Sport and Exercise Science, Faculty of Natural and Life Sciences, University of Salzburg, Salzburg, Austria; 10 Mental mHealth Lab, Department of Applied Psychology, Institute of Sports and Sports Science, Karlsruhe Institute of Technology, Karlsruhe, Germany; 11 Charité-Universitätsmedizin Berlin, Corporate Member of Freie Universität Berlin and Humboldt-Universität zu Berlin, NeuroCure Cluster of Excellence, Berlin, Germany

**Keywords:** Human behaviour, Addiction

## Abstract

Alcohol-related cues are thought to contribute to drinking in alcohol use disorder (AUD), but their contribution in daily life remains difficult to separate from changing social contexts and internal states. We used ecological momentary assessment in two independent cohorts of non-treatment-seeking individuals, comprising a discovery cohort with AUD (17–62 years; n = 73) and a young-adult replication cohort screened for risky drinking (19–35 years; n = 56), to test whether alcohol cue exposure was associated with momentary drinking after simultaneous multivariable adjustment for social context, affective states, and event appraisal. In logistic mixed-effects models, within-person exposure to alcohol-related cues was significantly and reproducibly associated with higher odds of drinking in both cohorts. Being in company, exposure to drinking companions, and higher energetic arousal also showed reproducible within-person associations with drinking. Exploratory clustering of participant-specific slopes suggested meaningful alcohol cue-reactive and social context-reactive risk profile candidates. These findings identify alcohol cue exposure as a robust daily-life correlate of drinking and suggest candidate phenotypes motivating future validation studies of more personalized, context-aware interventions for AUD.

## Introduction

Alcohol use disorder (AUD) is a significant contributor to the global burden of disease and mortality [1, 2]. Given that alcohol consumption is episodic and context-dependent, identifying correlates of drinking in daily life is essential [3]. Alcohol-related cues, in particular, are important proximal correlates of drinking [4, 5]. In incentive-sensitization and neurocircuitry models of addiction, such conditioned cues increase the likelihood of alcohol use by acquiring motivational salience, biasing attention, and evoking craving and approach behavior [6]. However, retrospective reports on drinking and related contexts are limited by recall bias and temporal aggregation [7].

Ecological momentary assessment (EMA) can address these limitations by capturing experiences and behaviors in daily life and real time [7, 8]. Indeed, previous real-world studies have linked daily or momentary exposure to alcohol cues with craving and drinking [9, 10]. However, these studies have typically examined narrower subsets of co-occurring influences. For example, Meredith et al. [9] examined cue exposure and mood states in once-daily retrospective reports of drinking quantity, whereas Kohen et al. [10] examined cue exposure, postdrinking status, and trait-level alcohol sensitivity with craving as the outcome. Additionally, social context [3, 11] and arousal [12] have also been linked to drinking in EMA research, but typically as focal predictors rather than as simultaneous covariates alongside cue exposure, affective states, and event appraisal. Accordingly, it remains unclear whether alcohol cue exposure shows a specific within-person association with momentary drinking when changing social context, affective states, arousal, and event appraisal are modeled simultaneously, and whether such an association replicates across independent cohorts.

Beyond average effects, alcohol cue-reactivity is heterogeneous across individuals. Laboratory and neuroimaging work has distinguished reward- versus relief-related drinking phenotypes with differing striatal cue-reactivity patterns [13] and appetitive, aversive, and mixed neural cue-reactivity subtypes [14]. This person-level variability suggests that the strength of within-person associations between daily-life predictors and drinking may itself define clinically meaningful risk profile candidates, a possibility that, to our knowledge, has not been examined using real-world behavioral data.

The present EMA study had two primary objectives, pursued in non-treatment-seeking individuals spanning risky drinking to AUD, and studied across two independent cohorts: a broader-age discovery cohort with on average mild-to-moderate AUD, and a young-adult replication cohort with risky drinking. First, we aimed to test and replicate the relationship between alcohol cue exposure and drinking likelihood after simultaneous multivariable within-person adjustment for co-occurring changes in social context, affective states, energetic arousal, and event appraisal in daily life. Second, we aimed to explore whether meaningful risk subtypes emerge from the covariance structure of individuals’ within-subject predictor-outcome associations.

## Methods

### Study design and participants

This work is a focused secondary analysis of EMA data collected across two funding periods of project A04 of the Collaborative Research Center TRR265, one of whose primary research interests is the study of the triggers of losing control over alcohol consumption, including alcohol cues (https://www.trr265.org/en). The discovery cohort was recruited during the first funding period, which focused on older individuals with mild-to-moderate AUD, whereas the replication cohort was recruited during the second funding period, in which the project was more specifically tailored to younger risky drinkers. The EMA protocol was modified during the second funding period in response to participant feedback regarding prompt timing and response format, and to enhance comparability with other similar research. Analyzing data across both funding periods was intended from the outset; because the data originate from these two successive protocols, the cohorts differ in assessment schedule, response formats for some items, and age range, making replication of the cue-drinking association across them a conservative test of robustness.

Participants were non-treatment-seeking individuals spanning risky drinking to AUD. The discovery cohort comprised adults with, on average, mild-to-moderate DSM-5 AUD (2–8 criteria), drawn from the TRR265 central cohort (projects A01/S01) and enrolled into the intensive 6-week EMA protocol, covering a broad age range (17–62 years). The replication cohort comprised young adults (19–35 years) recruited from the community (flyers, online, and website advertisements) and screened for risky drinking (AUDIT-C ≥ 5), with DSM-5 AUD criteria assessed subsequently at the in-person intake.

The discovery cohort was recruited in Mannheim between March 2021 and March 2023, and the replication cohort was recruited in Mannheim and Berlin between March 2025 and December 2025. General exclusion criteria included significant neurological conditions, substance use disorders other than alcohol, nicotine, or cannabis, head trauma, pregnancy, contraindications for magnetic resonance imaging, and a diagnosis of schizophrenia spectrum or bipolar disorders. Across both cohorts, 147 participants had available data; after EMA quality control (compliance > 25%), 129 were retained for analysis (discovery cohort, n = 73; replication cohort, n = 56; Table 1). Mean EMA compliance was 65.7 ± 20.1% in the discovery cohort and 66.5 ± 21.2% in the replication cohort. The within-person analyses were informed by 19,579 prompts across 129 participants, and the independent replication design provided an additional test of the primary cue-drinking association.

**Table 1:** Sample characteristics.

Characteristic	Discovery (n = 73)	Replication (n = 56)	p
Age, mean ± SD (range), years	37.71 ± 12.70 (17–62)	24.98 ± 3.89 (19–35)	<0.001
Sex, female/male, n (%)	28/45 (38.4%/61.6%)	27/29 (48.2%/51.8%)	0.285
AUDIT, mean ± SD (range)	13.67 ± 5.48 (3–26)	14.05 ± 6.71 (4–32)	0.729
Consumption intensity (sum of AUDIT items 1–3), mean ± SD (range)	6.27 ± 2.08 (2–12)	6.18 ± 1.88 (3–11)	0.785
DSM-5 AUD criteria, current, mean ± SD (range)	3.93 ± 1.56 (2–8)	3.45 ± 2.74 (0–11)	0.217
DSM-5 AUD criteria, lifetime, mean ± SD (range)	4.07 ± 2.32 (0–11)	4.89 ± 2.90 (0–11)	0.084
DSM-5 AUD severity, current (none/mild/moderate/severe), n (%)	0 (0%) / 31 (42.5%) / 30 (41.1%) / 12 (16.4%)	15 (26.8%) / 17 (30.4%) / 12 (21.4%) / 12 (21.4%)	
EMA compliance, mean ± SD, %	65.7 ± 20.1	66.5 ± 21.2	
Observations included, n	12,005	7574	

Current DSM-5 AUD severity categories were defined as none = 0–1, mild = 2–3, moderate = 4–5, and severe ≥ 6. The replication cohort was screened for risky drinking with the AUDIT-C ( ≥ 5) at recruitment, whereas DSM-5 AUD criteria were assessed at intake; accordingly, some replication participants do not meet current DSM-5 AUD criteria despite meeting the risky drinking screen. Between-cohort *p* values are from Welch’s *t*-tests for continuous variables and Fisher’s exact test for sex.

### Baseline assessments

Clinical and psychological assessment included basic sociodemographic information and a battery of established psychosocial self-report instruments, whose validated German versions were used. Alcohol-related baseline measures comprised current (last 12 months) and lifetime DSM-5 alcohol use disorder (AUD) criteria assessed using the Structured Clinical Interview for DSM-5 (SCID-5) [15, 16], the Alcohol Use Disorders Identification Test (AUDIT) [17] and a consumption-intensity index computed as the sum of AUDIT items 1–3. Stress-related constructs were assessed with the Social Readjustment Rating Scale (SRRS) [18] and the Perceived Stress Scale (PSS) [19], whereas perceived social support was assessed with the Oslo Social Support Scale (OSSS-3) [20]. Impulsivity was measured with the BIS-15 short form of the Barratt Impulsiveness Scale [21], and personality traits with the NEO Five-Factor Inventory dimensions of neuroticism, extraversion, openness, agreeableness, and conscientiousness [22–24]. Childhood adversity was operationalized using the Childhood Trauma Screener (CTS) and Childhood Trauma Questionnaire (CTQ)-based maltreatment domains covering emotional abuse, physical abuse, sexual abuse, emotional neglect, and physical neglect [25–27].

### EMA

EMA was administered on Android smartphones using MovisensXS. In the discovery cohort, which completed 6 weeks of EMA, weeks 1–3 comprised eight prompts per day: a morning prompt randomized within 08:00–09:00 and seven daytime prompts randomized within 09:00–22:00 with at least 1 h spacing between prompts. Weeks 4–6 comprised four prompts per day: a morning prompt randomized within 08:00–09:00 and three daytime prompts randomized within 09:00–22:00 with at least 2 h spacing between prompts. The replication cohort completed 4 weeks of EMA with seven prompts per day: a fixed morning prompt at 07:30 (answer window until 09:00), five daytime prompts randomized within 09:00–20:30 with at least 1 h spacing, and a fixed evening prompt at 21:00 (answer window until 22:30). All prompt types were combined and analyzed together.

The primary outcome was drinking at the moment, and the primary predictor was alcohol cue exposure since the last prompt. The cue stimuli comprised 11 alcohol-related images depicting alcoholic beverages and alcohol-present contexts and are shown in Supplementary Figure S2. Additional time-varying predictors were being in company, contact to people participants typically drink alcohol with, affective valence, energetic arousal, calmness, guiltiness, irritability, loneliness, anxiousness, and positive and negative event appraisal. For the affect and arousal items, the cohorts used different response formats and time frames (Table 2). Accordingly, these items were placed on a common analytic scale by SD-standardization of within-person deviations. Full item wording, response scales, and assessment windows are provided in Table 2.

**Table 2:** Primary EMA items.

EMA item/question	Scale/coding	Assessment window
Momentary drinking: “Are you drinking alcohol at the moment? [37]”	Binary yes/no	Momentary
Alcohol cue exposure: “Since the last prompt, have you been exposed to any of the drinks shown here or places where alcohol was present? [38]”	Binary yes/no^a^	Coverage since last prompt
Being in company: “Are you alone at the moment? [39]” (reverse-coded so 1 = in company)	Binary yes/no^b^	Momentary
Drinking companion exposure: “Since the last prompt, have you had contact with people you typically drink alcohol with?“	Binary yes/no	Coverage since last prompt
Affective valence, energetic arousal, and calmness (six bipolar items based on the MDBF) [40–42]	Discovery cohort: 1–7 Likert coverage ratings (“Since the last prompt, on average I felt …”); replication cohort: 0–100 visual analog momentary ratings (“At the moment, on average I feel …”); two items per dimension summed after reverse coding as needed	Discovery: coverage since last prompt; replication: momentary
Negative affect: “At the moment I feel …” irritated, lonely, guilty, and anxious [43]	1–7 Likert	Momentary
Positive and negative event appraisal [44]	VAS 0–100 ratings of the intensity of the most significant positive or negative event since the last prompt	Coverage since last prompt

Table 2 lists the primary EMA items entered into the multivariable models.

*MDBF* multidimensional mood questionnaire, *VAS* visual analog scale.

^a^The alcohol cue item referred to 11 alcohol-related images depicting alcoholic beverages and alcohol-present contexts; the cue set is shown in Supplementary Figure S2.

^b^In the replication cohort, virtual company was combined with the alone response for binarization.

### Statistical analysis

Momentary drinking was modeled separately in the discovery and replication cohorts using logistic mixed-effects models in R version 4.5.2. All time-varying predictors were decomposed into within-person deviations (person-mean centered) and between-person components (person means) and entered simultaneously into the models. Continuous within-person predictors were SD-standardized, and event appraisal ratings were log-transformed before decomposition. Models were adjusted for age, sex, and time of day and included a participant-specific random intercept; the replication-sample model additionally included site (Berlin/Mannheim). Odds ratios with 95% Wald confidence intervals were derived from the fixed-effect estimates. Model coefficients (β) and SEs are reported on their original 1-unit scale. For originally binary WP and PM predictors, ORs and CIs were rescaled to represent a 0.1-unit increase and were calculated as OR = exp(0.1β), providing a consistent reporting increment across these components. For binary WP predictors, a change from absence to presence corresponds to a 1-unit difference after person-mean centering, with OR = exp(β). This full contrast is additionally reported for the primary alcohol-cue predictor in the Results. Observations with missing values for any model term were excluded at the prompt level.

To address temporal ordering and potential co-occurrence of cue exposure and drinking, we performed two sensitivity analyses in both cohorts. In sensitivity analysis 1 (transition subset; same-time cue, prior non-drinking), we retained the primary model specification (all predictors at the current prompt, including alcohol cue exposure at the current prompt) but restricted the analyses to prompts preceded by non-drinking, thereby focusing on transitions into drinking and reducing the contribution of prompts in which reported cue exposure may merely reflect alcohol already present during a current drinking episode. In sensitivity analysis 2 (lagged cue; full sample), we replaced the within-person alcohol cue predictor at the current prompt with within-person cue exposure from the previous prompt (lagged), while keeping the remaining predictors at the current prompt; lagging was performed within participant and tested whether cue exposure at the prior prompt prospectively predicts drinking at the current prompt. All sensitivity models used the same fixed-effect structure as the primary GLMM, including the same within-person and between-person predictor decomposition; only the cue term (same-time vs lagged) or the observation subset differed. The results are summarized in Supplementary Table S4.

### Exploratory clustering and sensitivity analyses

To explore potential risk profiles, we refitted the model in the pooled sample and screened a fuller random-slopes specification that included participant-specific random slopes for the replicated within-person predictor-outcome associations, namely alcohol cue exposure, being in company, drinking companion exposure, and energetic arousal. After screening this random-slopes specification, the pooled model retained random slopes for alcohol cue exposure, being in company, and drinking companion exposure, whereas energetic arousal was not retained because of negligible slope variance. Participant-specific within-person random effects for alcohol cue exposure, being in company, and drinking companion exposure were extracted from the pooled-sample random-slopes model (see models’ specifications in Tables S2a-c). For each predictor, participant-specific slopes were computed as the sum of the fixed within-person effect and participant’s random slope. These three slope estimates were z-standardized across participants and used as input features for clustering. We applied k-means clustering [28] (Euclidean distance) to the standardized slope values. To guide the selection of the number of clusters, we examined solutions across a range of k values using the elbow criterion (total within-cluster sum of squares; Figure S1A) and average silhouette width [29] (Figure S1B). Based on these diagnostics, we selected k = 2 and retained the resulting cluster solution. Internal stability of the partition was characterized by participant-level bootstrap resampling and is reported in the Supplementary Information. To characterize the two clusters, we summarized descriptive statistics by cluster for the three participant-specific within-person slopes used in the clustering and for baseline sociodemographic and clinical variables. Between-cluster differences in continuous variables were tested using Welch’s t-tests; sex was compared using a chi-square test (Supplementary Table S3).

## Results

**Fig. 1:**
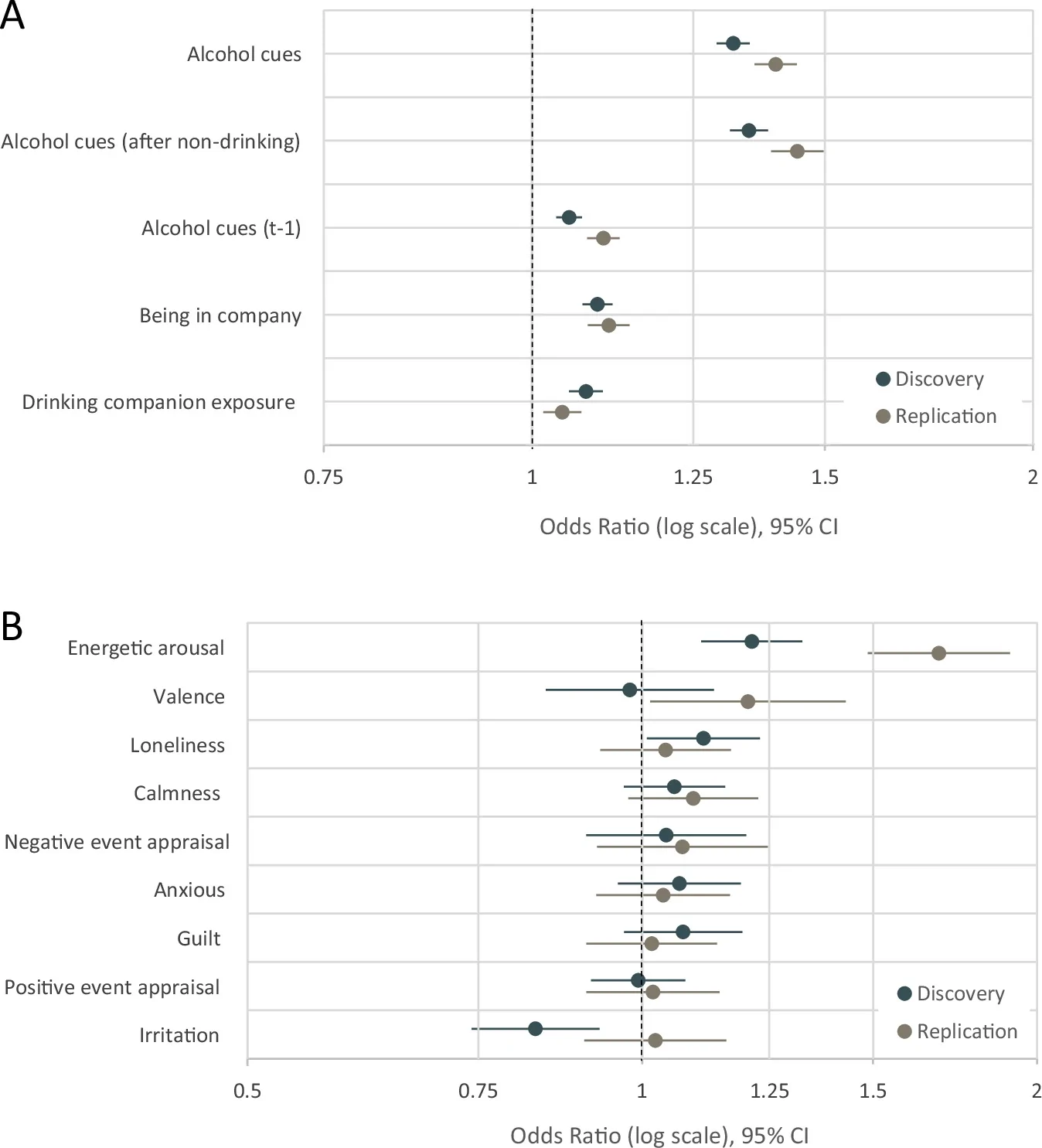
Within-person predictors of momentary drinking. **A** and **B** display within-person fixed effects from logistic mixed-effects models for the outcome of momentary drinking (yes/no). These effects are expressed as odds ratios (ORs), with 95% Wald confidence intervals (CIs) on a log scale. (**A**) shows binary predictors, and (**B**) shows continuous predictors. In (**A**), “Alcohol cues” denotes the primary same-time cue estimate, “Alcohol cues (after non-drinking)” denotes the same-time cue estimate restricted to prompts preceded by non-drinking (sensitivity analysis 1), and “Alcohol cues (t-1)” denotes the lagged cue estimate in which cue exposure at the previous prompt predicts current drinking (sensitivity analysis 2). Binary within-person predictors are reported per 0.1-unit increase, and continuous within-person predictors are standard deviation (SD)-standardized and reported per 1-SD increase. Points indicate model estimates for the discovery (dark blue) and replication (light brown) cohorts. For the complete model terms, including within-person and between-person terms, see Tables 3 and 4.

After adjusting for all other time-varying predictors, exposure to alcohol-related cues was significantly associated with momentary drinking in both the discovery cohort and the young-adult replication cohort (discovery OR = 1.32, 95% CI 1.29–1.35; replication OR = 1.40, 95% CI 1.36–1.44; both p < 0.001; Fig. 1A, Tables 3 and 4). These reported ORs use the 0.1-unit scale applied consistently to binary predictors throughout the manuscript. For a full 1-unit change from cue absent to cue present, the corresponding ORs were 16.19 (95% CI 12.90–20.33) in the discovery cohort and 29.17 (95% CI 21.76–39.11) in the replication cohort. This association remained significant even when cue exposure was lagged by one prompt or the analysis was restricted to prompts following non-drinking at the previous prompt (all p-values < 0.001, Fig. 1A, Supplementary Table S4).

**Table 3:** Discovery sample GLMM output.

Predictor	Estimate	SE	z	p	OR	95% CI
**Within-person (WP)**						
Alcohol cue exposure (WP)	2.784	0.116	23.988	<0.001	1.321	1.291–1.351
Being in company (WP)	0.908	0.106	8.592	<0.001	1.095	1.073–1.118
Drinking companion exposure (WP)	0.748	0.119	6.298	<0.001	1.078	1.053–1.103
Energetic arousal (WP, SD)	0.192	0.045	4.254	<0.001	1.212	1.109–1.325
Valence (WP, SD)	−0.022	0.075	−0.288	0.773	0.979	0.844–1.134
Loneliness (WP, SD)	0.108	0.051	2.125	0.034	1.113	1.008–1.230
Calmness (WP, SD)	0.042	0.072	0.588	0.557	1.043	0.906–1.200
Negative event appraisal (WP, SD)	0.057	0.045	1.249	0.212	1.058	0.968–1.157
Anxious (WP, SD)	0.065	0.055	1.184	0.237	1.067	0.958–1.189
Guilt (WP, SD)	0.072	0.053	1.357	0.175	1.075	0.969–1.192
Positive event appraisal (WP, SD)	−0.007	0.042	−0.168	0.866	0.993	0.914–1.079
Irritated (WP, SD)	−0.187	0.057	−3.265	0.001	0.829	0.741–0.928
**Between-person (PM)**						
Alcohol cue exposure (PM)	2.019	0.529	3.816	<0.001	1.224	1.103–1.357
Being in company (PM)	1.326	0.636	2.084	0.037	1.142	1.008–1.293
Drinking companion exposure (PM)	−1.859	0.525	−3.545	<0.001	0.830	0.749–0.920
Energetic arousal (PM, SD)	−0.305	0.174	−1.756	0.079	0.737	0.524–1.036
Valence (PM, SD)	−0.696	0.358	−1.946	0.052	0.498	0.247–1.005
Loneliness (PM, SD)	0.541	0.267	2.026	0.043	1.717	1.018–2.897
Calmness (PM, SD)	0.546	0.348	1.570	0.117	1.727	0.873–3.417
Negative event appraisal (PM, SD)	0.189	0.207	0.913	0.361	1.207	0.805–1.810
Anxious (PM, SD)	0.154	0.207	0.746	0.456	1.167	0.778–1.750
Guilt (PM, SD)	−0.381	0.208	−1.836	0.066	0.683	0.455–1.026
Positive event appraisal (PM, SD)	−0.237	0.185	−1.282	0.200	0.789	0.549–1.134
Irritated (PM, SD)	−0.757	0.299	−2.531	0.011	0.469	0.261–0.843
**Covariates**						
Age (Z-transformed)	0.443	0.121	3.657	<0.001	1.558	1.228–1.976
Female	−0.468	0.247	−1.890	0.059	0.626	0.386–1.017
Time of day (Z-transformed)	1.530	0.065	23.682	<0.001	4.619	4.070–5.243
Intercept	−4.834	0.297	−16.256	<0.001	0.008	0.004–0.014

Estimates and SEs are reported on the original 1-unit scale. ORs/CIs for originally binary WP/PM predictors are reported per 0.1-unit increase and were calculated as OR = exp(0.1 × Estimate); all other ORs/CIs are reported per 1-unit increase. n = 73 participants; 12005 observations.

*WP* within-person (person-mean-centered), *PM* person-mean (between-person), SD SD-standardized, *OR* odds ratio, *CI* confidence interval.

**Table 4:** Replication sample GLMM output.

Predictor	Estimate	SE	z	p	OR	95% CI
**Within-person (WP)**						
Alcohol cue exposure (WP)	3.373	0.150	22.551	<0.001	1.401	1.361–1.443
Being in company (WP)	1.063	0.148	7.198	<0.001	1.112	1.080–1.145
Drinking companion exposure (WP)	0.421	0.134	3.142	0.002	1.043	1.016–1.071
Energetic arousal (WP, SD)	0.521	0.064	8.169	<0.001	1.684	1.486–1.908
Valence (WP, SD)	0.186	0.088	2.120	0.034	1.204	1.014–1.429
Loneliness (WP, SD)	0.041	0.059	0.702	0.483	1.042	0.929–1.169
Calmness (WP, SD)	0.071	0.076	0.924	0.356	1.073	0.924–1.247
Negative event appraisal (WP, SD)	0.090	0.058	1.540	0.123	1.094	0.976–1.226
Anxious (WP, SD)	0.037	0.060	0.615	0.539	1.037	0.923–1.167
Guilt (WP, SD)	0.017	0.059	0.287	0.774	1.017	0.907–1.141
Positive event appraisal (WP, SD)	0.019	0.060	0.319	0.750	1.019	0.907–1.146
Irritated (WP, SD)	0.023	0.064	0.362	0.717	1.023	0.903–1.159
**Between-person (PM)**						
Alcohol cue exposure (PM)	5.008	0.955	5.246	<0.001	1.650	1.368–1.989
Being in company (PM)	0.333	0.850	0.392	0.695	1.034	0.875–1.221
Drinking companion exposure (PM)	−0.467	0.726	−0.643	0.520	0.954	0.828–1.100
Energetic arousal (PM, SD)	−0.057	0.188	−0.304	0.761	0.944	0.653–1.366
Valence (PM, SD)	0.573	0.363	1.577	0.115	1.773	0.870–3.615
Loneliness (PM, SD)	0.451	0.393	1.150	0.250	1.570	0.728–3.389
Calmness (PM, SD)	−0.491	0.360	−1.364	0.173	0.612	0.302–1.240
Negative event appraisal (PM, SD)	0.605	0.210	2.882	0.004	1.831	1.214–2.763
Anxious (PM, SD)	−0.319	0.222	−1.433	0.152	0.727	0.470–1.124
Guilt (PM, SD)	−0.496	0.362	−1.371	0.170	0.609	0.299–1.238
Positive event appraisal (PM, SD)	−0.369	0.220	−1.681	0.093	0.691	0.450–1.063
Irritated (PM, SD)	0.135	0.234	0.576	0.565	1.145	0.723–1.812
**Covariates**						
Age (Z-transformed)	0.342	0.199	1.715	0.086	1.407	0.952–2.080
Female	0.168	0.331	0.508	0.611	1.183	0.619–2.262
Site	−0.700	0.353	−1.982	0.047	0.497	0.249–0.992
Time of day (Z-transformed)	1.175	0.075	15.767	<0.001	3.238	2.798–3.748
Intercept	−5.463	0.464	−11.766	<0.001	0.004	0.002–0.011

Estimates and SEs are reported on the original 1-unit scale. ORs/CIs for originally binary WP/PM predictors are reported per 0.1-unit increase and were calculated as OR = exp(0.1 × Estimate); all other ORs/CIs are reported per 1-unit increase. n = 56 participants; 7574 observations.

*WP* within-person (person-mean-centered), *PM* person-mean (between-person), *SD* SD-standardized, *OR* odds ratio, *CI* confidence interval.

In addition, the time-varying predictors for being in company, energetic arousal, and exposure to drinking companions showed significant within-person associations with drinking in both cohorts (all p-values < 0.002, Figs. 1A and B, Tables 3 and 4). The remaining within-person predictors were not consistently associated with drinking (Fig. 1B, Tables 3 and 4).

**Fig. 2:**
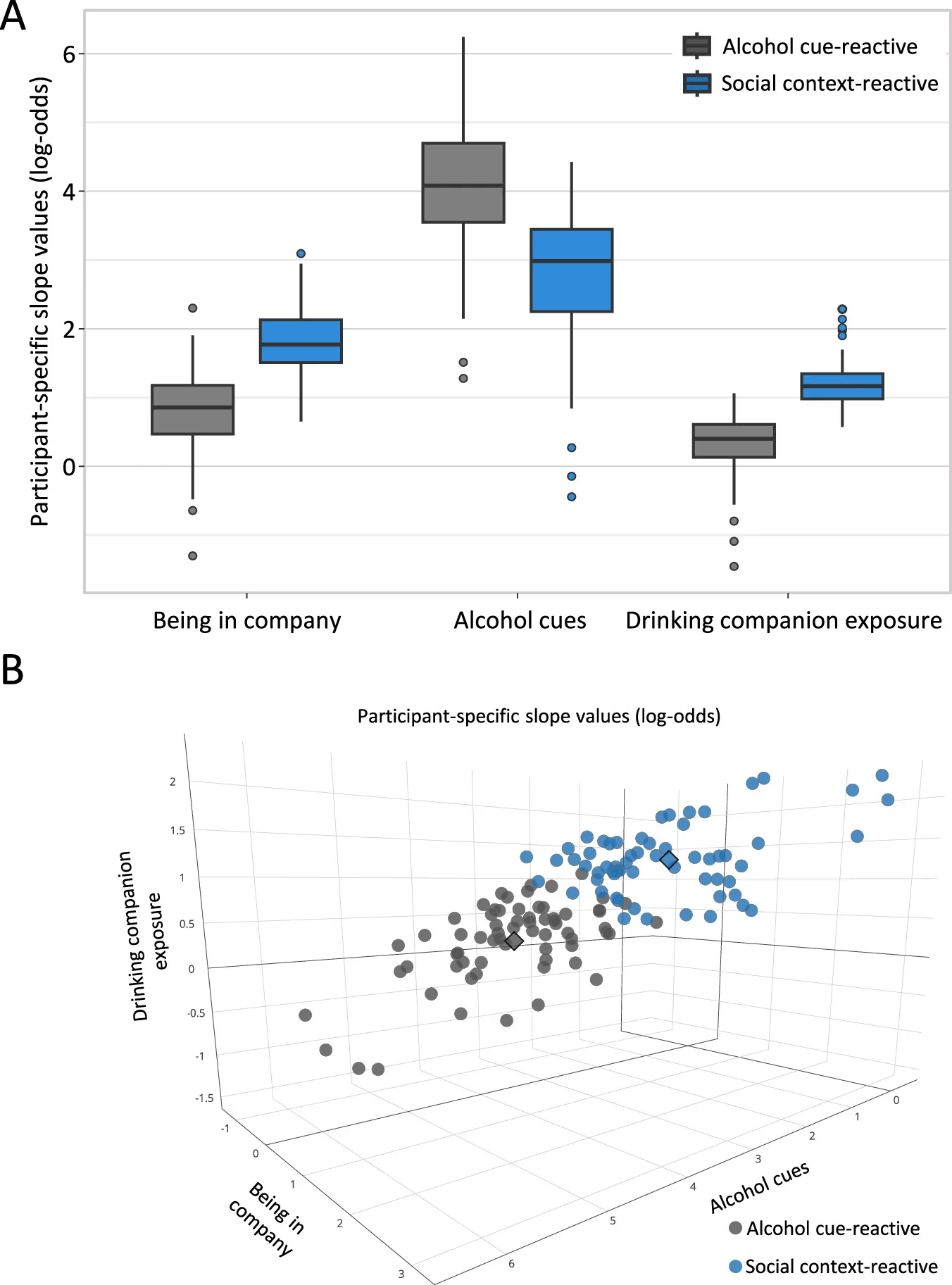
Cluster analysis of within-person random slope values. **A** displays the within-person random slope values (log-odds scale) for the two clusters for the predictors alcohol cue exposure, being in company, and drinking companion exposure, estimated from the pooled random-slopes model. Center lines indicate medians, boxes indicate Interquartile Ranges (IQRs). Whiskers extend 1.5 times the IQR from the top and bottom of the box. **B** shows that the individuals of both clusters map along an axis of alcohol cue-related versus social context-related drinking. Dots indicate within-person random slope values (log-odds scale), diamonds indicate cluster centroids. In both panels, dark gray colors depict the individuals assigned to the alcohol cue-reactive cluster, and blue colors depict those assigned to the social context-reactive cluster.

The clustering of participant-specific random slopes indicated a two-cluster solution (Fig. 2, Supplementary Figures S1 and S3). These clusters formed along an axis, splitting individuals into two groups: one group for which drinking was rather related to alcohol cues, and another group for which it was rather related to social context variables (Fig. 2B). Post-hoc comparisons showed that the individuals in the alcohol cue-reactive cluster were significantly older, met more DSM-5 criteria for AUD, had higher total AUDIT scores, and experienced more stressful life events than those in the social context-reactive cluster (Supplementary Table S3).

## Discussion

In two independent cohorts of non-treatment-seeking individuals spanning risky drinking to AUD, exposure to alcohol cues showed a reproducible, within-person association with real-world alcohol consumption under simultaneous multivariable adjustment. As such, our data provide real-world evidence consistent with a central prediction of the incentive sensitization theory [30]: that exposure to alcohol cues may be linked to alcohol use in daily life, even when social context, affective states, and event appraisal are taken into account. The findings extend prior real-world studies linking cue exposure and cue-related craving to near-term use and relapse [9, 10, 31].

Beyond alcohol cues, social context and energetic arousal showed replicable within-person associations with drinking. This aligns with social facilitation and opportunity models [3] and EMA studies linking social context [11] and higher arousal [12] to increased drinking. In contrast, negative affective states were not reproducibly associated with alcohol consumption, consistent with meta-analytic evidence that daily affect-drinking associations are heterogeneous and often smaller than commonly assumed [32].

Our findings further suggested candidate profiles of daily-life alcohol consumption, with a cue-reactive profile comprising relatively older and more severely affected individuals and a social context-reactive profile comprising relatively younger and less severely affected individuals. These patterns are consistent with broader literature on heterogeneity in alcohol cue-reactivity, including reward- versus relief-related drinking phenotypes with distinct clinical and neural correlates [13] and appetitive versus aversive cue-reactivity subtypes [14]. The cue-reactive profile may be more comparable with relief- or severity-related drinking, whereas the social context-reactive profile may be more compatible with reward- and socially motivated drinking [13, 33]. Although the present design cannot speak to treatment response, such daily-life phenotyping may, if validated, complement efforts to match individuals to mechanism-targeted interventions [33].

Together, these observations raise the hypothesis that the relative importance of conditioned motivational processes versus social opportunity for drinking may vary across age and disease stages [3, 34]. The applied daily-life methods may help facilitate the differentiation of these processes, and, if validated in prospective studies, the resulting insights may inform the development of future cue- and context-focused interventions [8].

## Limitations

Several limitations should be considered. First, the reported within-person associations are correlational and observational; despite the temporal-ordering sensitivity analyses, they cannot establish that cue exposure causally increases drinking. Second, the cluster analysis was exploratory, and k-means imposes a partition on participant-specific slopes that are continuously distributed and estimated with empirical-Bayes shrinkage, so the resulting groups should be regarded as approximate, hypothesis-generating poles that require independent validation. Third, the two cohorts did not use identical affect and arousal assessments; the items differed in response format and time frame and were placed on a common analytic scale by within-person SD-standardization, so the larger energetic-arousal OR in the replication cohort should not be over-interpreted as evidence of a true cross-cohort difference. Finally, there is theoretical concern that frequent EMA may induce measurement reactivity [7]. Although prior work suggests that such effects are often absent, or otherwise modest or short-lived in substance-use monitoring studies [35, 36], and our within-person, person-mean-centered models absorb stable individual-level shifts, measurement reactivity cannot be fully excluded.

## Conclusions

Alcohol cue exposure shows a replicable association with real-world drinking. The exploratory alcohol cue- versus social context-reactive risk profiles may, upon future validation, help with the development of more personalized targets for daily-life interventions in AUD.

## Supplementary information

supplementary information

## Data Availability

Sharing of data is possible upon reasonable request and within the defined limitations of the EU general data protection laws.

## Code availability

The custom code used for the analyses of this study is available from the corresponding author upon reasonable request.
