# Supplementary material for: Real-world alcohol cue exposure and drinking risk in alcohol use disorder
Source: Transl Psychiatry. 2026 Oct 8;16:537. doi: 10.1038/s41398-026-04506-4 (PMC13649650; doi:10.1038/s41398-026-04506-4)
Supplement: Supplementary file 1 [file 41398_2026_4506_MOESM1_ESM.docx]

**SUPPLEMENTAL INFORMATION**

**Real-world alcohol cue exposure and drinking risk in alcohol use disorder**

**Authors:** Ali Ghadami^1,8^, MSc, Abhijit Sreepada^1,8^, MSc, Iris Reinhard^2^, PhD, Marvin Ganz^1^, MSc, Justin Boehmer^3,9,10^, PhD, Kanya Meyer-Degering^3,9^, MSc, Henrik Walter^9,10^, MD PhD, Jamila Andoh^1^, PhD, Bernd Lenz^5,8^, MD, Patrick Bach^5,8^, MD PhD, Falk Kiefer^5,8^, MD PhD, Emanuel Schwarz^1,4,8^, PhD, Markus Reichert^1,6^, PhD, Ulrich W. Ebner-Priemer^1,7^, PhD, Andreas Meyer-Lindenberg^1,8^, MD PhD, Christine Heim^3,10,11^, PhD, Heike Tost^1,8^, MD PhD

^1^ Department of Psychiatry and Psychotherapy, Central Institute of Mental Health, University of Heidelberg, Medical Faculty Mannheim, Mannheim, Baden-Württemberg, Germany.

^2^ Core Facility Biostatistics, Central Institute of Mental Health, University of Heidelberg, Medical Faculty Mannheim, Mannheim, Baden-Württemberg, Germany.

^3^ Charité-Universitätsmedizin Berlin, Corporate Member of Freie Universität Berlin and Humboldt-Universität zu Berlin, Institute of Medical Psychology, Berlin, Germany.

^4^ Hector Institute for Artificial Intelligence in Psychiatry, Central Institute of Mental Health, Medical Faculty Mannheim, Heidelberg University, Mannheim, Germany.

^5^ Department of Addictive Behavior and Addiction Medicine, Central Institute of Mental Health, Medical Faculty Mannheim, Heidelberg University, Mannheim, Germany.

^6^ Department of Sport and Exercise Science, Faculty of Natural and Life Sciences, University of Salzburg, Salzburg, Austria.

^7^ Mental mHealth Lab, Department of Applied Psychology, Institute of Sports and Sports Science, Karlsruhe Institute of Technology, Karlsruhe, Germany

^8^ German Center for Mental Health (DZPG), Partner Site Mannheim-Heidelberg-Ulm, Germany

^9^ Charité-Universitätsmedizin Berlin, Corporate Member of Freie Universität Berlin and Humboldt-Universität zu Berlin, Department of Psychiatry and Psychotherapy (CCM), Berlin, Germany.

^10^ German Center for Mental Health (DZPG), Partner Site Berlin-Potsdam, Germany

^11^ Charité-Universitätsmedizin Berlin, Corporate Member of Freie Universität Berlin and Humboldt-Universität zu Berlin, NeuroCure Cluster of Excellence, Berlin, Germany.

Table of Contents

[1. Supplementary Information	3](#_Toc237605816)

[Multilevel analysis	3](#_Toc237605817)

[Bootstrap cluster-stability analysis	4](#_Toc237605818)

[2. Supplementary Tables	5](#_Toc237605819)

[Table S1. Pooled sample GLMM output	5](#_Toc237605820)

[Table S2a. Random effects variance estimates from full model	7](#_Toc237605821)

[Table S2b. Model fit statistics for pooled-sample random-effects specifications	8](#_Toc237605822)

[Table S2c. Random effects variance estimates from primary (reduced) model	9](#_Toc237605823)

[Table S3. Exploratory clustering descriptives and post hoc comparisons	9](#_Toc237605824)

[Table S4. Sensitivity analyses: Cue effect estimates	10](#_Toc237605825)

[3. Supplementary Figures	12](#_Toc237605826)

[Figure S1. Cluster analysis diagnostics	12](#_Toc237605827)

[Figure S2. Alcohol-related cue images	14](#_Toc237605828)

[Figure S3. Individual slopes (log-odds) for the three predictor pairs, by cluster.	16](#_Toc237605829)

[4. Supplementary References	17](#_Toc237605830)

# 1. Supplementary Information

## Multilevel analysis

Continuous WP deviations were SD-standardized, and binary predictors (alcohol cue exposure, being in company, and drinking companion exposure) were coded as 0/1 at each prompt and then decomposed into person means (PM; proportions) and within-person deviations (WP; deviations from the person mean) for modeling. For pooled analysis, SD-standardization of continuous within-person deviations was performed in the combined dataset to ensure a common scale across cohorts. All models were adjusted for age, sex, and time of day. Observations with missing values for any model term were excluded (complete case at the observation level). Event-appraisal intensity ratings were log-transformed prior to the WP/PM decomposition. Analyses were conducted in R version 4.5.2 ^1^ using lme4::glmer, with estimation via maximum likelihood (Laplace approximation) using the bobyqa optimizer and increased iteration limits.

**Model specification.** For participant *i* at prompt *j*, the primary model can be written as

**(1)**

$$\text{logit}\left( P(\text{drinking}_{ij}=1) \right)=\beta_{00}+\sum_{k\in\mathcal{K}_{\text{WP}}} \beta_{k0}X_{k,ij}^{\text{WP}}+\sum_{k\in\mathcal{K}_{\text{PM}}} \beta_{0k}X_{k,i}^{\text{PM}}+\beta_{020}\text{ }\text{age}_{i}+\beta_{030}\text{ }\text{female}_{i}+\beta_{040}\text{ }\text{time}_{ij}+u_{0i}$$

Where the within-person predictor set $\mathcal{K}_{\text{WP}}$comprised: valence (WP, SD), positive event appraisal (WP, SD), negative event appraisal (WP, SD), being in company (WP), alcohol cue exposure (WP), drinking companion exposure (WP), anxious (WP, SD), loneliness (WP, SD), irritated (WP, SD), guilt (WP, SD), energetic arousal (WP, SD), calmness (WP, SD) and the between-person predictor set $\mathcal{K}_{\text{PM}}$comprised: valence (PM), positive event appraisal (PM, SD), negative event appraisal (PM, SD), being in company (PM), alcohol cue exposure (PM), drinking companion exposure (PM), anxious (PM, SD), loneliness (PM, SD), irritated (PM, SD), guilt (PM, SD), energetic arousal (PM, SD), calmness (PM, SD).

$P(\text{drinking}_{ij}=1)$ is the probability that participant *i* reported currently drinking at assessment *j*. Within-subject effects were modelled at Level 1 (EMA prompts within individuals) and between-subject effects at Level 2 (differences between individuals). For each EMA predictor, the WP component equals the observed value minus participant *i*’s mean for that predictor, and the PM component equals participant *i*’s mean for that predictor. Fixed effects include the intercept $\beta_{00}$, WP and PM main effects, and covariate effects (age, female, time of day). Random effects in the cohort-specific primary models included a participant-specific intercept only.

**Effect size reporting.** Odds ratios (OR) were computed as $\text{OR}=\exp(\hat{\beta})$, with Wald 95% confidence intervals $\exp(\hat{\beta}\pm1.96\cdot SE(\hat{\beta}))$. For originally binary WP and PM predictors, ORs/CIs were rescaled to represent a 0.1-unit increase and were calculated as $\text{OR}=\exp\left( 0.1\hat{\beta} \right)$. For binary WP predictors, a change from absence to presence corresponds to a 1-unit difference after person-mean centering, with $\text{OR}=\exp(\hat{\beta})$. ORs/CIs for all other predictors were reported per 1-unit increase (continuous WP terms are SD-standardized). Wald z-tests were used for fixed effects. Model convergence and diagnostics were evaluated using gradient tolerance and the number of function evaluations (maxfun = 2e6); convergence was confirmed for all reported models.

**Discovery/replication/pooled sample workflow.** The model was fitted in the discovery sample, re-estimated with the same specification plus site (Mannheim, Berlin, reference = Mannheim) as a covariate in the replication sample, and then re-estimated in the pooled dataset where both site (same coding) and cohort (discovery, replication; reference = discovery) were included as covariates. To identify predictors with substantial between-participant heterogeneity for the replicated items (alcohol cue exposure (WP), being in company (WP), drinking companion exposure (WP), and energetic arousal (WP)), we first fitted a random-slopes screening model including random slopes for these four within-person predictors (Full model). This model showed a singular fit, due to the negligible heterogeneity for energetic arousal (WP). Accordingly, the primary pooled model retained a random intercept and random slopes only for three predictors of alcohol cue exposure (WP), being in company (WP), drinking companion exposure (WP) (Reduced model; Table S2c), dropping slopes with negligible variance for parsimony and model stability. Model fit statistics for the Full vs Reduced specifications are reported in Table S2b.

## Bootstrap cluster-stability analysis

To characterize the internal stability of the exploratory two-cluster partition, we generated 500 bootstrap samples by resampling with replacement from the 129 participant-level, z-standardized slope vectors and reran k-means clustering in each sample (*k* = 2; 100 random starts). Cluster stability was quantified using cluster-specific Jaccard similarity and the partition-level adjusted Rand index (ARI). For each reference cluster, Jaccard similarity was calculated with the best-matching bootstrap cluster among the uniquely sampled participants. For the ARI, all participants in the original sample were assigned to their nearest centroid from each bootstrap solution, and the resulting partition was compared with the reference partition. The cue-reactive and social context-reactive reference clusters comprised 67 and 62 participants, respectively. Mean Jaccard coefficients were 0.949 and 0.944, respectively, and the mean ARI was 0.894 (SD = 0.090). These findings indicate high internal stability of the partition under resampling of the participant-level slope features. However, these results do not constitute independent validation of distinct subtypes.

# 2. Supplementary Tables

## Table S1. Pooled sample GLMM output

| **Predictor** | **Estimate** | **SE** | **z** | **p** | **OR** | **95% CI** |
| --- | --- | --- | --- | --- | --- | --- |
| **Within-person (WP)** | | | | | | |
| Alcohol cue exposure (WP) | 3.522 | 0.228 | 15.458 | <.001 | 1.422 | 1.360 - 1.487 |
| Being in company (WP) | 1.365 | 0.171 | 7.973 | <.001 | 1.146 | 1.108 - 1.185 |
| Drinking companion exposure (WP) | 0.765 | 0.143 | 5.358 | <.001 | 1.080 | 1.050 - 1.110 |
| Energetic arousal (WP, SD) | 0.370 | 0.042 | 8.759 | <.001 | 1.447 | 1.333 - 1.572 |
| Valence (WP, SD) | 0.095 | 0.056 | 1.682 | .093 | 1.099 | 0.984 - 1.228 |
| Loneliness (WP, SD) | 0.082 | 0.041 | 1.994 | .046 | 1.085 | 1.001 - 1.176 |
| Calmness (WP, SD) | 0.050 | 0.050 | 1.005 | .315 | 1.052 | 0.953 - 1.160 |
| Negative event appraisal (WP, SD) | 0.057 | 0.037 | 1.552 | .121 | 1.059 | 0.985 - 1.138 |
| Anxious (WP, SD) | 0.030 | 0.043 | 0.687 | .492 | 1.030 | 0.946 - 1.122 |
| Guilt (WP, SD) | 0.025 | 0.042 | 0.604 | .546 | 1.025 | 0.945 - 1.112 |
| Positive event appraisal (WP, SD) | 0.027 | 0.036 | 0.752 | .452 | 1.027 | 0.958 - 1.101 |
| Irritated (WP, SD) | -0.097 | 0.043 | -2.246 | .025 | 0.907 | 0.834 - 0.988 |
| **Between-person (PM)** | | | | | | |
| Alcohol cue exposure (PM) | 3.134 | 0.552 | 5.672 | <.001 | 1.368 | 1.228 - 1.524 |
| Being in company (PM) | 1.126 | 0.556 | 2.026 | .043 | 1.119 | 1.004 - 1.248 |
| Drinking companion exposure (PM) | -1.169 | 0.474 | -2.469 | .014 | 0.890 | 0.811 - 0.976 |
| Energetic arousal (PM, SD) | -0.107 | 0.137 | -0.783 | .434 | 0.898 | 0.686 - 1.175 |
| Valence (PM, SD) | 0.117 | 0.267 | 0.439 | .661 | 1.124 | 0.667 - 1.896 |
| Loneliness (PM, SD) | 0.002 | 0.188 | 0.010 | .992 | 1.002 | 0.693 - 1.449 |
| Calmness (PM, SD) | -0.081 | 0.249 | -0.325 | .745 | 0.922 | 0.567 - 1.501 |
| Negative event appraisal (PM, SD) | 0.165 | 0.157 | 1.050 | .294 | 1.179 | 0.867 - 1.605 |
| Anxious (PM, SD) | -0.058 | 0.149 | -0.386 | .699 | 0.944 | 0.705 - 1.264 |
| Guilt (PM, SD) | -0.040 | 0.161 | -0.250 | .802 | 0.961 | 0.701 - 1.317 |
| Positive event appraisal (PM, SD) | -0.171 | 0.154 | -1.106 | .269 | 0.843 | 0.623 - 1.141 |
| Irritated (PM, SD) | 0.130 | 0.192 | 0.679 | .497 | 1.139 | 0.782 - 1.660 |
| **Covariates** | | | | | | |
| Age (Z-transformed) | 0.302 | 0.105 | 2.880 | .004 | 1.352 | 1.101 - 1.660 |
| Female | -0.268 | 0.208 | -1.288 | .198 | 0.765 | 0.509 - 1.150 |
| Time of day (Z-transformed) | 1.391 | 0.050 | 27.621 | <.001 | 4.018 | 3.641 - 4.435 |
| Site | -0.446 | 0.338 | -1.319 | .187 | 0.640 | 0.330 - 1.242 |
| Cohort | 0.363 | 0.219 | 1.659 | .097 | 1.437 | 0.936 - 2.207 |
| Intercept | -6.396 | 0.446 | -14.328 | <.001 | 0.002 | 0.001 - 0.004 |

Note: WP = within-person (person-mean-centered); PM = person-mean (between-person); SD = SD-standardized; OR = odds ratio; CI = confidence interval. Estimates and SEs are reported on the original 1-unit scale. ORs/CIs for originally binary WP/PM predictors are reported per 0.1-unit increase and were calculated as $\text{OR}=\exp\left( 0.1\hat{\beta} \right)$; all other ORs/CIs are reported per 1-unit increase. Random slopes included for being in company (WP), alcohol cue exposure (WP), and drinking companion exposure (WP). n = 129 participants; 19579 observations.

## Table S2a. Random effects variance estimates from full model

Random-slope variance estimates from the pooled-sample maximal random-slopes screening model, including random slopes for four within-person predictors (being in company, alcohol cue exposure, drinking companion exposure, and energetic arousal). This table documents the empirical basis for simplifying the random-effects structure in the primary pooled model.

| **Predictor** | **Variance** | **SD** | **Retained in Reduced?** |
| --- | --- | --- | --- |
| Intercept | 1.855 | 1.362 | Yes |
| Being in company (WP) | 1.309 | 1.144 | Yes |
| Alcohol cue exposure (WP) | 2.853 | 1.689 | Yes |
| Drinking companion exposure (WP) | 0.851 | 0.923 | Yes |
| Energetic arousal (WP, SD) | 0.053 | 0.231 | No |

**Note:** WP = within-person (person-mean-centered); SD = SD-standardized

## Table S2b. Model fit statistics for pooled-sample random-effects specifications

Model fit indices for the pooled-sample maximal random-slopes screening model and primary reduced random effects model.

| **Model** | **Random effects** | **AIC** | **BIC** | **logLik** | ***N* observations** | ***N* participants** |
| --- | --- | --- | --- | --- | --- | --- |
| Full model | Random intercept + random slopes for being in company (WP), alcohol cue exposure (WP), drinking companion exposure (WP), energetic arousal (WP, SD) | 6234.9 | 6589.6 | -3072.4 | 19579 | 129 |
| Reduced model | Random intercept + random slopes for being in company (WP), alcohol cue exposure (WP), drinking companion exposure (WP) | 6241.5 | 6556.8 | -3080.7 | 19579 | 129 |

**Note:** AIC/BIC/logLik values are reported as printed in the model summaries; both models were fit using maximum likelihood (Laplace approximation).

## Table S2c. Random effects variance estimates from primary (reduced) model

| **Effect** | **Variance** | **SD** | **Correlations (with intercept; between slopes)** |
| --- | --- | --- | --- |
| (Intercept) | 1.748 | 1.322 | - |
| Being in company (WP) | 1.353 | 1.163 | corr with intercept = -0.35 |
| Alcohol cue exposure (WP) | 2.836 | 1.684 | corr with intercept = -0.46; corr with company = -0.26 |
| Drinking companion exposure (WP) | 0.776 | 0.881 | corr with intercept = -0.32; corr with company = 0.45; corr with cues = -0.54 |

## Table S3. Exploratory clustering descriptives and post hoc comparisons

|  | C1 (n = 67) mean ± SD / Count | C2 (n = 62) mean ± SD / Count | Welch t / chi-square | t_df | p (t) /  p (χ²) |
| --- | --- | --- | --- | --- | --- |
| **Individual Slopes** | | | | | |
| Being in company (WP) | 0.77 ± 0.66 | 1.86 ± 0.50 | -10.62 | 122.88 | <.001 |
| Alcohol cue exposure (WP) | 4.01 ± 0.99 | 2.70 ± 1.09 | 7.14 | 123.58 | <.001 |
| Drinking companion exposure (WP) | 0.31 ± 0.46 | 1.19 ± 0.38 | -11.92 | 125.60 | <.001 |
| Sociodemographic | | | | | |
| Age | 37.09 ± 12.49 | 26.89 ± 8.02 | 5.56 | 113.54 | <.001 |
| Sex | Female: 27 / Male: 40 | Female: 28 / Male: 34 | 0.14 | 1 | .704 |
| Income | 4.62 ± 2.42 | 3.20 ± 2.37 | 3.33 | 123.27 | .001 |
| Education | 7.06 ± 1.47 | 6.92 ± 2.15 | 0.43 | 102.78 | .665 |
| Psychological/Clinical assessments | | | | | |
| AUD criteria | 4.28 ± 2.27 | 3.11 ± 1.86 | 3.22 | 125.17 | .002 |
| AUD criteria lifetime | 5.01 ± 2.63 | 3.79 ± 2.45 | 2.74 | 126.99 | .007 |
| AUDIT | 14.91 ± 6.24 | 12.68 ± 5.60 | 2.14 | 126.89 | .034 |
| Consumption intensity (sum of AUDIT items 1-3) | 6.55 ± 2.23 | 5.89 ± 1.63 | 1.94 | 120.68 | .054 |
| SRRS | 361.47 ± 192.05 | 270.41 ± 165.45 | 2.87 | 124.40 | .005 |
| CTS | 7.81 ± 3.05 | 7.21 ± 2.43 | 1.23 | 124.36 | .220 |
| PSS | 27.03 ± 7.12 | 26.82 ± 6.37 | 0.18 | 124.88 | .861 |
| OSSS-3 | 10.26 ± 2.14 | 10.44 ± 2.00 | -0.50 | 124.98 | .615 |
| BIS15 | 33.35 ± 8.07 | 33.12 ± 7.04 | 0.17 | 118.67 | .868 |
| neo_Neuroticism | 10.61 ± 5.24 | 9.70 ± 5.27 | 0.97 | 124.13 | .336 |
| neo_Extraversion | 14.11 ± 3.65 | 14.80 ± 3.76 | -1.06 | 123.51 | .292 |
| neo_Openness (to experience) | 14.11 ± 5.20 | 14.07 ± 5.18 | 0.04 | 124.32 | .965 |
| neo_Agreeableness | 15.83 ± 4.55 | 16.93 ± 3.80 | -1.49 | 123.78 | .140 |
| neo_Conscientiousness | 15.98 ± 4.82 | 17.44 ± 3.93 | -1.87 | 123.14 | .063 |
| CTQ_emotional_abuse | 10.09 ± 5.03 | 9.00 ± 4.67 | 1.28 | 127.00 | .204 |
| CTQ_physical_abuse | 6.52 ± 2.51 | 5.74 ± 1.78 | 2.05 | 119.30 | .043 |
| CTQ_sexual_abuse | 5.91 ± 2.21 | 5.45 ± 1.17 | 1.49 | 101.95 | .139 |
| CTQ_emotional_neglect | 12.07 ± 5.44 | 9.27 ± 4.20 | 3.28 | 123.14 | .001 |
| CTQ_phys_neglect | 9.43 ± 3.58 | 8.50 ± 2.64 | 1.69 | 121.17 | .093 |

**Note:** C1 = alcohol cue-reactive cluster, C2 = social context-reactive cluster, WP = within-person slopes (log-odds scale). Clusters were derived via k-means clustering (k = 2) of z-standardized participant-specific slopes. Between-cluster comparisons used Welch's t-tests for continuous variables and a chi-square test for sex; df values are from Welch's t-tests.

## Table S4. Sensitivity analyses: Cue effect estimates

Alcohol cue coefficient and odds ratio from Supplementary models in discovery and replication samples. All models are logistic GLMMs with the same fixed-effect specification as the primary model; only the cue term (same-time vs lagged) and/or the observation subset differs.

| **Cohort** | **Model description** | **n (participant / obs)** | **Estimate** | **SE** | **z** | **p** | **OR** | **95% CI** |
| --- | --- | --- | --- | --- | --- | --- | --- | --- |
| Discovery | same-time cue; subset: drinking at t-1 = 0 | 73 / 10850 | 3.004 | 0.134 | 22.441 | <.001 | 1.350 | 1.315 - 1.386 |
| Discovery | lagged cue (t-1) (full sample) | 73 / 11932 | 0.515 | 0.091 | 5.675 | <.001 | 1.053 | 1.034 - 1.072 |
| Replication | same-time cue; subset: drinking at t-1 = 0 | 56 / 6831 | 3.673 | 0.185 | 19.828 | <.001 | 1.444 | 1.392 - 1.497 |
| Replication | lagged cue (t-1) (full sample) | 56 / 7518 | 0.990 | 0.114 | 8.662 | <.001 | 1.104 | 1.080 - 1.129 |

**Note:** WP = within-person (person-mean-centered); OR = odds ratio; CI = confidence interval (Wald). Estimate/SE/z are on the original log-odds scale. “Lagged” = cue at previous prompt (*t*−1) within the same participant. ORs and 95% CIs represent a 0.1-unit increase and were calculated as $\text{OR}=\exp\left( 0.1\hat{\beta} \right)$.

# 3. Supplementary Figures

## Figure S1. Cluster analysis diagnostics

Diagnostics for selecting the number of clusters (k-means) for exploratory clustering based on participant-specific within-person slopes

**Figure S1A.** Elbow plot.

**Figure S1B.** Silhouette plot.

## Figure S2. Alcohol-related cue images

## Figure S3. Individual slopes (log-odds) for the three predictor pairs, by cluster.

Figure S3A: Alcohol cues vs Being in company.

Figure S3B: Alcohol cues vs Drinking companion exposure.

Figure S3C: Being in company vs Drinking companion exposure.

# 4. Supplementary References

1	R Core Team. R: A Language and Environment for Statistical Computing (R Foundation for Statistical Computing, Vienna, Austria, 2025).
